# Association Analysis of Single Nucleotide Polymorphisms at Five Loci: Comparison between Atopic Dermatitis and Asthma in the Chinese Han Population

**DOI:** 10.1371/journal.pone.0035334

**Published:** 2012-04-24

**Authors:** Hua-Yang Tang, Xian-Fa Tang, Xian-Bo Zuo, Jin-Ping Gao, Yu-Jun Sheng, Yang Li, Fu-Sheng Zhou, Xian-Yong Yin, Feng-Li Xiao, Wei-Dong Du, Sen Yang, Liang-Dan Sun, Xue-Jun Zhang

**Affiliations:** 1 Institute of Dermatology and Department of Dermatology, No.1 Hospital, Anhui Medical University, Hefei, Anhui, China; 2 Key Laboratory of Dermatology, Anhui Medical University, Ministry of Education, China, Hefei, Anhui, China; 3 State Key Laboratory Incubation Base of Dermatology, Anhui Medical University, Hefei, Anhui, China; Maastricht University Medical Center, The Netherlands

## Abstract

Atopic diseases, such as atopic dermatitis (AD) and asthma, are closely related to clinical phenotypes with hypersensitivity, and often share some similar genetic and pathogenic bases. Our recent GWAS identified three susceptibility gene/loci *FLG* (rs11204971 and rs3126085), 5q22.1 (rs10067777, rs7701890, rs13360927 and rs13361382) and 20q13.33 (rs6010620) to AD. The effect of these AD associated polymorphisms in asthma is so far unknown. To investigate whether AD relevant genetic variants is identical to asthma and reveal the differences in genetic factors between AD and asthma in Chinese Han population, seven AD associated single nucleotide polymorphisms (SNPs) as well as 3 other SNPs (rs7936562 and rs7124842 at 11q13.5 and rs4982958 at 14q11.2) from our previous AD GWAS were genotyped in 463 asthma patients and 985 controls using Sequenom MassArray system. We found rs4982958 at 14q11.2 was significantly associated with asthma (*P* = 3.04×10^−4^, OR = 0.73). We also detected one significant risk haplotype GGGA from the 4 SNPs (rs10067777, rs7701890, rs13360927 and rs13361382) at 5q22.1 in AD cases (*P*_correction_ = 3.60×10^−10^, OR = 1.26), and the haplotype was suggestive of risk in asthma cases in this study (*P* = 0.014, *P*_correction_ = 0.084, OR = 1.38). These SNPs (rs11204971, rs3126085, rs7936562, rs712484 and rs6010620) at AD susceptibility genes/loci *FLG*, 11q13.5 and 20q13.33 were not associated with asthma in this study. Our results further comfirmed that 14q11.2 was an important candidate locus for asthma and demonstrated that 5q22.1 might be shared by AD and asthma in Chinese Han population.

## Introduction

Atopic diseases, such as atopic dermatitis (AD), asthma and allergic rhinitis (AR), are closely related to clinical phenotypes that are often observed with hypersensitivity. The mechanisms leading to atopic diseases development remain yet unknown. It is supposed that the cases would attribute to the interacted effect of multiple genetic variations and environmental factors on their pathophysiology. Asthma is documented being associated with atopic dermatitis [1]. Approximately half of AD patients, particularly in the severe AD, will develop into asthma [2].

Previous linkage study, candidate gene and GWAS have identified several susceptibility loci/genes predisposing to AD and asthma. Some common genetic factors such as *FLG*, *ADAM33*, and *GSDML/ORMDL3* had been observed in patients with AD and asthma [3]. Other genetic investigations illuminated several important candidate genes in Chromosome 14q11.2, which could contribute to the genetic predisposition to specific allergic reactions characterized by asthma and high level of serum IgE [4], [5]. In one previous GWAS of AD in the European populations, 11q13.5 (*C11orf30*) was highlighted as a susceptibility locus of AD [6], which was also pointed to contain a concurrent involvement of the common genetic factors for AD and asthma [7]. In one recent AD GWAS, we identified two novel susceptibility loci 5q22.1 (rs10067777, rs7701890, rs13360927 and rs13361382), 20q13.33 (rs6010620) and confirmed susceptibility gene *FLG* (rs11204971 and rs3126085) of AD [8]. The genetic effect of these AD associated factors in asthma is unknown.

In this study, we further analyze the 7 SNPs described above at *FLG*, 5q22.1, 20q13.33, as well as 3 other SNPs (rs7936562 and rs7124842 at 11q13.5, rs4982958 at 14q11.2) to identify genetic variants for asthma and reveal the common and different genetic factors for AD and asthma in the Chinese Han population.

## Methods

### Subjects

A total of 463 patients with asthma and 985 controls were enrolled in this study (Table 1). All samples were unrelated and Chinese Han in origin. Patients with asthma were recruited from No. 1 Hospital of Anhui Medical University in China according to the American Thoracic Society (ATS) guidelines [9]: First, the presence of at least two of the following symptoms was examined: recurrent cough, wheezing or dyspnea. Second, we examined for increased airway responsiveness to methacholine or the presence of reversible airflow limitation; the latter refers to 15% variability in the forced expiratory volume in 1 second (FEV1), or in the peak expiratory flow rate with or without an inhaled short-acting β2-agonist. Third, there was the absence of any other pulmonary diseases. Clinical information was collected from the affected individuals through a full clinical checkup by physician specialists. Additional demographic information was collected from both cases and controls through a structured questionnaire. All controls were clinically assessed to be without asthma, other atopic diseases, family history of atopic diseases (including first-, second- and third-degree relatives) or systemic disorders. The study was approved by the Institutional Ethics Committee of Anhui Medical University and was conducted according to Declaration of Helsinki principles. Collection of blood samples and clinical information from cases and controls was undertaken after written informed consent was obtained from all participants. DNA was extracted from peripheral blood lymphocytes using QIAamp DNA Blood kit (Qiagen, Valencia, CA, USA) according to the manufacturer's instructions. The extracted genomic DNAs were analyzed by agarose gel electrophoresis, quantified by spectrophotometer, and stored at −80°C until used.

**Table 1:** Summary information of asthma patients and health controls.

	Cases	Controls
Total number	463	985
Age(years)	9.76±8.52	25.83±11.83
Male (%)	273(59.0%)	560(56.9%)
Female (%)	190(41.0%)	425(43.1%)
with AD	49(10.6%)	NA
with AR	153(33.0%)	NA

NA = Not available.

### SNP selection and genotyping

We selected 10 SNPs based on our previous AD GWAS and genotyped them in 463 asthma patients. Seven of the 10 SNPs were associated with AD (rs11204971 and rs3126085 in *FLG*, rs10067777, rs7701890, rs13360927 and rs13361382 at 5q22.1, and rs6010620 at 20q13.33). Additional 2 top SNPs within 11q13.5 (rs7936562 and rs7124842) and one top SNP within 14q11.2 (rs4982958) in discovery samples of our AD GWAS were also selected. The annotated nominal gene was taken from the Illumina SNP database. SNPs were genotyped using the Sequenom MassArray system at State Key Laboratory Incubation Base of Dermatology, Ministry of National Science and Technology, Hefei, Anhui, China. Approximately 15 ng of genomic DNA was used to genotype each sample. Locus-specific PCR and detection primers were designed using the MassARRAY Assay Design 3.0 software (Sequenom). The DNA samples were amplified by multiplex PCR reactions, and the PCR products were then used for locus-specific single-base extension reactions. The resulting products were desalted and transferred to a 384-element SpectroCHIP array. Allele detection was performed using MALDI-TOF MS. The mass spectrograms were analyzed by the MassARRAY Typer software (Sequenom).

### Statistical analysis

The SNPs were analyzed for association with the disease by means of comparison of the minor allele frequency in the cases and the controls using PLINK 1.07 software [10]. The level of associated significance was assigned at *P* values of less than 0.005 after Bonferroni Multiple Testing correction for asthma case-control analysis, and less than 5.0×10^−8^ for AD GWAS. In addition to the allelic test of association, the genetic models (dominant model, recessive model and additive model) were calculated for the associated SNPs. For the SNPs at 5q22.1 (*TMEM232/SLC25A46*), haplotype analyses were performed to calculate haplotype frequencies and significance of associations for asthma case-control study by using Haploview v4.2 [11]. The extent of genetic heterogeneity was assessed by using Cochran's Q test and the I^2^ index [12] between AD and asthma groups. All the SNPs passed the quality control in terms of with a call rate >95%, Hardy–Weinberg equilibrium (HWE) (P>0.01) in the controls. The genetic statistical power for all genotyped SNPs was estimated using CaTS-Power Calculator software [13].

## Results

SNP rs4982958 at 14q11.2 was significantly associated with asthma in this study (*P* = 3.04×10^−4^, OR = 0.73). Risk allele C of the SNP rs4982958 represented the major allele and allele T was protective one in Chinese Han population for asthma (Table 2). The genotype TT appeared significantly less frequent in asthma cases (5.5%) than in controls (12.9%), higher frequency of the genotype CC was observed in cases (46.1%) than in controls (39.8%). The difference in distribution of rs4982958 genotype between the asthma cases and controls was statistically significant (*P* = 5.96×10^−5^) (Table 3). Those SNPs locating within AD susceptibility gene/loci *FLG*, 5q22.1, 11q13.5 and 20q13.33 did not reach the threshold significant association for asthma (*P*>0.005, after Bonferroni Multiple Testing correction) (Table 2). For the SNPs in *FLG* (rs11204971 and rs3126085) and 14q11.2 (rs4982958), we found significant evidence for genetic heterogeneity between AD and asthma groups in this study (*P*_het_≤0.0031, I^2^≥88.6) (Table 2).

**Table 2:** Summary of association results of 10 SNPs in five loci/genes and heterogeneity test between AD and asthma groups.

SNP	Chr	Gene	Allele^a^	Association evidence in AD GWAS	Association result in Asthma					Heterogeneity test	
					MAF^b^		*P* c	OR (95% CI)	Statistical power	*P*_het_ d	I^2^(%)^e^
					Cases	Controls					
rs11204971	1q21.3	*FLG*	A/G	susceptibility gene	0.4556	0.4220	8.96E−02	1.15 (0.98–1.34)	15%	0.0008	91.18
rs3126085	1q21.3		G/A		0.4446	0.4202	2.18E−01	1.10 (0.94–1.29)	6%	0.0014	90.25
rs7701890	5q22.1	*TMEM232/SLC25A46*	G/A	susceptibility loci	0.1296	0.1249	7.22E−01	1.04 (0.83–1.32)	1%	0.2763	15.62
rs10067777	5q22.1		G/A		0.1231	0.1133	4.45E−01	1.10 (0.86–1.40)	2%	0.5265	0
rs13360927	5q22.1		G/A		0.1247	0.1233	9.15E−01	1.01 (0.80–1.29)	1%	0.2313	30.20
rs13361382	5q22.1		A/G		0.1278	0.1214	6.42E−01	1.06 (0.83–1.36)	1%	0.4284	0
rs7124842	11q13.5	*C11orf30*	A/G	susceptibility gene	0.2516	0.2813	9.62E−02	0.86 (0.72–1.03)	13%	0.4422	0
rs7936562	11q13.5		A/G		0.4143	0.4354	2.87E−01	0.92 (0.78–1.08)	4%	0.9393	0
rs4982958	14q11.2	*CMA1*	T/C	no evidence	0.2969	0.3656	3.04E−04	0.73 (0.62–0.87)	78%	0.0031	88.60
rs6010620	20q13.33	*TNFRSF6B/ZGPA*	G/A	susceptibility loci	0.2613	0.2585	8.74E−01	1.02 (0.85–1.21)	1%	0.1369	54.81

a Minor allele/major allele.

b MAF, minor allele frequency.

c Two tail test *P* value.

d *P*_het_: *P* value of Cochran's Q test between AD group and asthma group.

e The I^2^ index describes the proportion of the total variability that is due to heterogeneity as described in reference 12.

**Table 3:** Distribution of genotypes and genetic model analysis for rs4982958 in asthma patients and controls.

	Cases(n = 458)	Controls(n = 982)	OR (95% CI)	*P*
Genotype				
CC	211(46.1%)	391(39.8%)	Reference	5.69E−05^a^
TT	25(5.5%)	127(12.9%)	0.37(0.23–0.58)	
TC	222(48.4%)	464(47.3%)	0.89(0.70–1.12)	
Recessive model				
TT/(TC+CC)	25(5.5%)/433(94.5%)	127(12.9%)/855(87.1%)	0.39(0.25–0.61)	3.05E−05
Dominant model				
(TT+TC)/CC	247(53.9%)/211(46.1%)	591(60.2%)/391(39.8%)	0.77(0.62–0.97)	2.50E−02
Additive model				
TT/TC/CC	25(5.5%)/222(48.4%)/211(46.1%)	127(12.9%)/464(47.3%)/391(39.8%)	0.60(0.48–0.76)	1.75E−05

T:Minor allele(effect allele), C:Major allele(reference allele).

a Patients vs controls using 2×3 contingency table.

Six haplotypes in combination with the 4 SNPs (rs10067777, rs7701890, rs13360927 and rs13361382) were established at 5q22.1 in asthma samples, and 3 haplotypes in AD samples. The integrated haplotype GGGA showed a significant association with AD (*P*_correction_ = 3.60×10^−10^, OR = 1.26) and weakly significant in asthma cases and controls (*P* = 0.014, OR = 1.38) (Table 4), which had no genetic heterogeneity between AD and asthma (*P*_het_ = 0.590, I^2^ = 0). After Bonferroni Multiple Testing correction, haplotype GGGA was no more significant for asthma (*P*_correction_ = 0.084) (Table 4).

**Table 4:** Haplotype analysis of 4 SNPs within 5q22.1 in AD/asthma patients and controls.

Haplotype	Asthma cases-controls					AD cases-controls					Heterogeneity test	
	F_A	F_U	OR (95%CI)	*P*	*P*_correction_ a	F_A	F_U	OR (95%CI)	*P*	*P*_correction_ a	*P*_het_ b	I^2^(%)^c^
AAAG	0.859	0.837	1.21(0.94–1.46)	0.126	0.756	0.837	0.864	0.90(0.85–0.96)	1.85×10^−3^	5.55×10^−3^	0.089	65.35
GGGA	0.114	0.086	1.38(1.07–1.78)	0.014	0.084	0.139	0.093	1.26(1.17–1.35)	1.20×10^−10^	3.60×10^−10^	0.590	0
AGGA	0.001	0.023	0.05(0.01–0.33)	1.08×10^−5^	6.48×10^−5^	N/A	N/A	N/A	N/A	N/A	N/A	N/A
GAAG	0.003	0.024	0.13(0.04–0.43)	4.70×10^−5^	2.82×10^−4^	N/A	N/A	N/A	N/A	N/A	N/A	N/A
AGAG	0.008	0.014	0.63(0.28–1.38)	0.198	1	N/A	N/A	N/A	N/A	N/A	N/A	N/A
AAGA	0.008	0.012	0.64(0.27–1.50)	0.329	1	0.009	0.018	0.86(0.68–1.10)	0.213	0.639	0.085	66.29

a *P*_correction_: *P* value after Bonferroni Multiple Testing correction.

b *P*_het_: *P* value of Cochran's Q test between AD group and asthma group.

c The I^2^ index describes the proportion of the total variability that is due to heterogeneity as described in reference 12.

## Discussion

AD and asthma are complex genetic diseases, which would result from integrated effect of multiple genetic and environmental factors on pathophysiology process of the entities. Some common genetic factors being shared in the patients with AD and asthma have been identified [3]. Here we investigated the association of 10 SNPs within *FLG*, 5q22.1, 11q13.5, 14q11.2 and 20q13.33 for asthma in the Chinese Han population, to evaluate the relationship between AD and asthma in these loci/genes.

**Figure 1:**
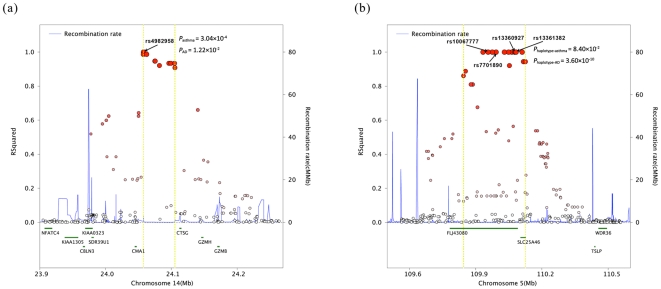
Regional LD plots for associated SNPs. The r^2^ values (y-axis) of SNPs were plotted against their map positions (x-axis). Estimated recombination rates (based on the combined CHB and JPT samples from the HapMap project) were plotted in light blue. Gene annotations were adapted from the University of California at Santa Cruz Genome Browser (http://genome.ucsc.edu/). (a): Regional LD plots for rs4982958 at 14q11.2. (b): Regional LD plots for rs10067777, rs7701890, rs13360927 and rs13361382 at 5q22.1.

At the locus 14q11.2, the five genes: *KIAA0323*, *SDR39U1*, *CMA1*, *CTSG* and *GZMH* were located within a single 220 kb LD block surrounding the tag biomarker rs4982958 which was associated with asthma in this study (Figure 1a). Previous studies selected mast cell chymase gene (*CMA1*) at 14q11.2 as a candidate gene for analysis of AD, asthma and other allergic phenotypes [14]. Mast cell tryptase and chymase are key players in mediating chronic inflammation, as observed in asthmatics of the lungs. Two polymorphisms in *CMA1*, including promoter polymorphism rs1800875 (−1903G/A) [15]–[18] and (TG)n(GA)m repeat polymorphism [19]–[21], were identified being associated with the allergic phenotypes in different populations. However, other studies failed to replicate role of −1903G/A (rs1800875) in AD or asthma [18], [22]–[24]. Inconsistence in these studies probably was attributable to different populations, variation on phenotype definitions, ethnicity and environmental exposure.

We observed that the association of rs4982958 with asthma under additive model (*P* = 1.75×10^−5^) was of more significance than that under dominant model (*P* = 2.50×10^−2^) and recessive model (*P* = 3.05×10^−5^) by using a logistic regression analysis (Table 3). Clearly, risk allele C of rs4982958 was the major one and allele T was of protection effect. Compared with C/C, the genotype of homozygote T/T (OR_Hom_ = 0.37) showed smaller OR than the heterozygote T/C (OR_Het_ = 0.89) (Table 3). This result may provide more information for futher study on this locus. Rs4982958 located at 10.4 kb upstream of *CMA1*. Linkage disequilibrium (LD) test showed that the two SNPs (rs4982958 and rs1800875) were moderately correlated with each other (D' = 1.0, r^2^ = 0.5) in CHB and JPT from 1000 Genomes Project data. Imputation of promoter polymorphism rs1800875 of *CMA1* was performed in our AD GWAS samples and then tested for association (*P* = 1.11×10^−2^, OR = 0.85). In AD GWAS, rs4982958 at 14q11.2 was replicated in expanded AD samples, we found a weak evidence of association in the Chinese Han population (*P* = 1.22×10^−2^, OR = 0.94), but did not reach genome-wide significance (P<5.0×10^−8^) [8]. Statistical tests showed that the odds ratio of rs4982958 in asthma samples (OR = 0.73) was smaller than in AD (OR = 0.94) and the existence of significant genetic heterogeneity between AD and asthma (*P*_het_ = 0.0031, I^2^ = 88.60) in Chinese Han population.

Our AD GWAS had identified 5q22.1 as a susceptibility locus for AD [8]. In this study, further haplotype analysis of the 4 SNPs (rs10067777, rs7701890, rs13360927 and rs13361382) within 5q22.1 was performed in AD and asthma groups, respectively. Only two haplotypes (AAAG and GGGA) had allele frequencies >5% (Table 4). The risk haplotype GGGA had a consistent association evidence for AD (*P*_correction_ = 3.60×10^−10^, OR = 1.26) as single SNP did (Table 4). It further supported the presence of underlying causal variants within this locus for AD. None of the 4 SNPs within 5q22.1 was associated with asthma in such a small samples size and low statistical power (1%∼2%) in this study. The risk allele of these SNPs at this locus was the same for AD and asthma. In asthma group, haplotype GGGA was a suggestive risk factor in this study (*P* = 0.014), although it was barely below significance (*P* = 0.084) after multiple testing correction. We observed that the odds ratio of haplotype GGGA (OR = 1.38) was much higher than single SNP in asthma cases-controls samples (1.04≤OR≤1.10). Further more, heterogeneity test at 5q22.1 showed that no genetic heterogeneity was found for each SNPs (0.2313≤*P*_het_≤0.5265, 0≤I^2^≤30.2) (Table 2) and risk haplotype GGGA (*P*_het_ = 0.590, I^2^ = 0) (Table 4) between AD and asthma groups, which means 5q22.1 might have similar effect on AD and asthma. The four SNPs (rs10067777, rs7701890, rs13360927 and rs13361382) located within a 600 kb LD block harboring two genes *TMEM232* (*FLJ43080*) and *SLC25A46*. Little is known about the function of the two genes. At 5q22.1, *TSLP* and *WDR36* were proposed to be candidate genes for asthma in Caucasian population [25]–[27]. Nevertheless, we found haplotype GGGA fall into different LD blocks with *TSLP* and *WDR36*, which were separated by a recombination hot spot (Figure 1b). Fine-mapping or direct sequencing would be powerful to further search for any potential and associated susceptibility genes at 5q22.1 dedicated to both AD and asthma.

The 5 SNPs in *FLG*, 11q13.5 and 20q13.33 did not reached the statistical significance in this study, which might either result from low statistical power (1%∼15%) (Table 2) or be attributed to 10.6% of these asthma cases with previous history of AD (Table 1). Comprehensive sequencing of entire *FLG* in asthma patients and larger scale of asthma samples or increased related tagSNPs for 11q13.5 and 20q13.33 would reliably enable to explore the relationship between AD and asthma in these loci/gene.

In conclusion, we compared the identical SNPs from the asthma cohort and our previous AD GWAS. We comfirmed 14q11.2 was an important candidate locus for asthma, and also demonstrated that 5q22.1 might be shared by AD and asthma in Chinese Han population. However, the evidences for the associations will be required to validate in diverse populations and then gain a better understanding on AD and asthma pathogenesis.
